# Restoring Tissue Homeostasis at Metastatic Sites: A Focus on Extracellular Vesicles in Bone Metastasis

**DOI:** 10.3389/fonc.2021.644109

**Published:** 2021-03-22

**Authors:** Domenica Giannandrea, Valentina Citro, Elena Lesma, Monica Bignotto, Natalia Platonova, Raffaella Chiaramonte

**Affiliations:** Department of Health Sciences, Università degli Studi di Milano, Milano, Italy

**Keywords:** metastasis, extracellular vesicles, therapeutic targets, metastatic niche, bone, osteoclast, osteoblast, mesenchymal stromal cell

## Abstract

Bone is the most common site of cancer metastasis and the spread of cancer cells to the bone is associated with poor prognosis, pain, increased risk of fractures, and hypercalcemia. The bone marrow microenvironment is an attractive place for tumor dissemination, due to the dynamic network of non-malignant cells. In particular, the alteration of the bone homeostasis favors the tumor homing and the consequent osteolytic or osteoblastic lesions. Extracellular vesicles (EVs) are reported to be involved in the metastatic process, promoting tumor invasion, escape from immune surveillance, extravasation, extracellular matrix remodeling, and metastasis, but the role of EVs in bone metastases is still unclear. Current results suggest the ability of tumor derived EVs in promoting bone localization and metastasis formation, altering the physiological balance between bone destruction and new bone depositions. Moreover, EVs from the bone marrow niche may support the onset of tumor metastasis. This review summarizes recent findings on the role of EVs in the pathological alterations of homeostasis that occur during bone metastasis to show novel potential EV-based therapeutic options to inhibit metastasis formation.

## Introduction

### Extracellular Vesicles: Biogenesis and Characteristics

Extracellular vesicles (EVs) are lipidic membrane-delimited nanoparticles released by all cell types. They are involved in physiologic and pathologic cellular communication. EVs represent a heterogeneous group of particles defined by size, shape, density, internal cargo, surface molecules, membrane components, cellular origin, and function (1).

According to their biogenesis and size, EVs are classified in exosomes (30–100 nm); microvesicles (MVs) (100–1000 nm), and apoptotic bodies (> 2000 nm) (1, 2).

The biogenesis of exosomes starts with the active process of endocytosis, followed by the formation of early endosomes and the maturation of late endosomes or multivesicular bodies (MVBs). Exosomes are derived from the invagination of the MVB membrane and the formation of intraluminal vesicles (ILVs) (3, 4). This process is regulated by the endosomal sorting complex required for transport (ESCRT) (4), although an ESCTR-independent process involving tetraspanins (5, 6) or sphingolipid ceramide (7) has been described. Mature MVBs can be directed to the degradation in lysosomes or fuse with the plasma membrane to release ILVs in the extracellular space as exosomes (4). The process of MVB fusion with the plasma membrane and exosome secretion requires the recruitment of soluble N-ethylmaleimide-sensitive factor attachment protein receptors (SNAREs), synaptotagmin-7, Rabs, and other Ras GTPases (1, 8).

The biogenesis of MVs still requires better characterization. MVs are generated through the budding of the plasma membrane and their consequent release in the extracellular compartment (2). This event involves the redistribution of membrane proteins and lipids leading to changes in membrane stiffness and the final transfer of the molecular cargo in the MV lumen (9). MV biogenesis requires ESCRT along with Ras GTPase in addition to the ADP-ribosylation factor 6 (ARF6) (9). ARF6 is involved in MV key features, including the selective recruitment of MV cargo, and the remodeling of the actin cytoskeleton necessary for MV release (10).

EV membrane composition is important for internalization in receiving cells. Indeed, the uptake mechanism may depend on proteins and glycoproteins located at the surface of both vesicles and target cells (11). EV internalization requires clathrin-dependent endocytosis or clathrin-independent mechanisms including caveolin-mediated uptake, macropinocytosis, phagocytosis, and lipid raft-mediated internalization (11). EVs exploit these mechanisms to transfer molecular messengers such as proteins, metabolites, lipids, mRNAs, and microRNAs (miRNAs) to target cells (12), affecting the functions and phenotypes of recipient cells by altering gene expression or by activating various signaling pathways (2).

### Bone Metastasis

Despite improvements in the treatment of primary tumors, patients often develop a metastatic disease that involves the bone. Bone is the most common site of metastasis (13, 14), and the site of disease that produces the greatest morbidity. Skeletal morbidity includes pain, that may require radiotherapy, hypercalcemia, pathologic fracture, and spinal cord or nerve root compression. It represents a complication mostly of solid tumors such as advanced breast, prostate, lung cancer, gastrointestinal, genitourinary, and thyroid carcinoma and, to a lesser extent, melanoma (15, 16). Moreover, the diffusion of skeletal lesions in multiple myeloma (MM) patients represents the formation of multiple bone metastases, too (17). MM, breast, and prostate cancer contributes to up to 70% of bone metastases cases (18). Approximately 70% of patients that die from breast and prostate cancers have bone metastases (14) and more than 80% of MM patients show skeletal lesions (19).

Bone is an attractive site for tumor dissemination due to the presence of the supportive and dynamic BM niche, composed of different interconnected hematopoietic and non-hematopoietic cell populations, which include immune cells, osteoclasts (OCLs), mesenchymal stromal cells (MSCs), osteoblasts (OBLs), adipocytes, chondrocytes, fibroblasts and endothelial cells (ECs) (20). These cell populations cooperate to sustain the normal bone homeostasis through a complex network of molecular signals including cytokines and growth factors or mediated by direct cell contact (21).

In physiological conditions, the balance between OCL-mediated bone degradation and bone mineralization carried out by OBLs is regulated by the bidirectional ephrin2-ephB4 signaling activated by direct cellular interaction (22). Moreover, OBLs can either stimulate, by cytokines such as receptor activator of nuclear factor k-B ligand (RANKL) and macrophage colony-stimulating factor (M-CSF), or inhibit by osteoprotegerin (OPG) or osteoclastogenesis inhibitory factor, OCL differentiation (23). In the regulation of bone remodeling, the endothelium plays a key role, too. Indeed, experiments using an EC specific, tamoxifen-inducible *Cdh5(PAC)-CreERT2* and *Rosa26-mT/mG* Cre reporter transgenic mice indicated that vessels with high expression of CD31 and endomucin may support osteoprogenitors in the perivascular niche (24). In turn, an osteoporotic mouse model showed that during bone remodeling, pre-OCLs release factors able to induce vessel recruitment such as the platelet derived growth factor-BB (PDGF-BB) (25). Also, immune cells participate in bone homeostasis regulating bone formation or osteoclastogenesis (26). For instance, T cells inhibit osteoclastogenesis (27, 28), but in the presence of inflammatory stimuli, they release RANKL and tumor necrosis factors (TNFα), promoting bone resorption (29–31).

The formation of bone metastasis begins with the detachment of cancer cells from the primary tumor; the invasion of surrounding tissue and the intravasation in the circulating system (20). Bone localization is favored by the release of chemoattractants, among which the CX-Chemokine ligand 12 (CXCL12) is the most relevant signal present in the bone marrow (BM) stroma (32–34).

Circulating tumor cells that escape immune surveillance, may finally reach BM vessels, extravasate, and establish a favorable and supportive microenvironment that initially sustains micrometastasis onset and, later, their expansion to form macrometastasis and invade the surrounding tissue (35).

The alteration of normal bone homeostasis is crucial for metastasis engraftment and depending on the different types of tumor, results in osteolytic, osteoblastic, or mixed lesions. The outcome depends on the mechanism activated by tumor cells to interfere with normal bone remodeling, and may result in bone destruction, new bone formation, or both.

The osteolytic metastasic process is associated with two key events in metastasis formation, the organization of a hospitable premetastatic niche for cancer cell settlement and the subsequent micrometastasis expansion.

In the osteolytic metastatic process, the interaction between the tumor cells and the BM niche leads to a “vicious cycle” (36), where bone metastatic tumor cells produce cytokines and growth factors which stimulate directly OCL maturation or indirectly induce the release of IL-6 and RANKL by OBLs or BM stromal cells (BMSCs) (16, 37). The degraded bone matrix is a source of tumor growth factors that lead to tumor proliferation, survival, and invasion, creating an endless loop involved in the formation of osteolytic lesions (16, 20).

In osteoblastic metastasis, high levels of OBLs provide support to tumor cell proliferation and survival. The WNT signaling originated from tumor cells is essential to direct OBL differentiation by activating the transcription factor RUNT-related transcription factor 2 (RUNX2) (21). Other factors involved are fibroblast growth factor (FGF), bone morphogenetic proteins (BMPs), Insuline-like growth factor (IGF), endothelin-1, and transforming growth factor (TGF)-β (36).

The alteration of bone homeostasis results in clinical complications involving pathologic fractures, bone pain, hypercalcemia and, spinal cord compression (18, 38), which affect the patient’s quality of life and survival, hampering a successful therapeutic strategy.

Despite recent therapeutic improvements, bone metastases are still incurable and to find effective novel therapeutic targets is an urgent issue.

#### Bone Homeostasis Alteration in Metastasis: A New Role for EVs

In the alteration of bone homeostasis associated with the metastatic process, EVs are new emerging players. Here, we report current evidence regarding the role of EVs in BM homeostasis dysregulation, including, on one side, the promotion of key events in metastasis engraftment together with the migration of tumor cells to the bone, the preparation of the pre-metastatic niche, and the onset of the metastasis, and on the other, the role of EVs derived from the bone metastatic niche in supporting tumor cells growth and survival.

##### EVs Promote Tumor Cell Recruitment at the Metastatic Site and Premetastatic Niche Formation

EVs have been reported to support the dissemination of tumor cells to distant sites, promoting the migration and the alteration of the premetastatic niche (39).

Angiogenesis, extracellular matrix remodeling, and immunosuppression promote the formation of the premetastatic niche by preparing the microenvironment at distant sites for the colonization of circulating tumor cells. Depending on the type of primary cancer cells, primary tumors, and shed cancer EVs can reprogram or educate target cells, contributing to metastatic organotropism.

The exosomes released by metastatic cells can enhance the invasive capacity. The intravenous injection of CD105-positive renal cell carcinoma-derived EVs enhances the expression of pro-angiogenic factors such as vascular endothelial growth factor (VEGF), matrix metalloproteinase-2 (MMP-2), and MMP-9 and activates angiogenic phenotypes in normal human endothelial cells, stimulating their growth and vessel formation in SCID mice (40). EVs can enhance endothelial permeability and promote tumor cell trans-endothelial migration. Triple-negative breast cancer (TNBC) cell lines can release miR-939 in exosomes, downregulating VE-cadherin at the level of adherent junctions in endothelial cells HUVECs, thereby favoring tumor cell intravasation (41). Furthermore, EVs are capable of increasing the metastatic potential of tumor cells. The high miR-223 levels in platelet-secreted EVs from non-small-cell lung carcinoma (NSCLC) patients reduce the protein level of the tumor suppressor EPB41L3, promoting lung cancer cell invasion (42).

EVs may increase bone metastasis favoring primary tumor cells dissemination at this site. For instance, *in vitro* experiments have shown that bone-derived soluble factors not only elicited the chemotactic response of osteotropic melanoma cells, but promoted their ability to reprogram non-osteotropic melanoma cells to express CXCR7 and, consequently, acquire bone osteotropism mediated by the CXCL-12 chemotactic gradient (43).

Tumor-derived EVs can also induce microenvironment modification. The miRNA-940 delivered by prostate cancer cells-derived EVs or its overexpression in breast cancer cell-derived EVs can induce OBL differentiation, causing bone lesions (44, 45). Prostate cancer-derived exosomes mediate the transfer of pyruvate kinase M2 (PKM2) in BMSCs modulating their behavior. PKM2 acts as a co-activator of HIF-1α-mediated transcription, enhancing CXCL12 production, and increasing BMSC ability to recruit tumor cells by expressing the chemokine receptor CXCR4 (46). Furthermore, *in vitro* prostate cancer cells communicate with OBLs through EV-associated RNA cargo enhancing OBL viability and creating a supportive environment for prostate cancer progression (47). Similarly, exosomes from melanoma cells can reprogram BM progenitor cells, inducing them to exit the BM and migrate to subsequent metastatic sites, such as the lungs, conditioning the local site toward a pro-vasculogenic environment and thereby promoting the homing and out-growth of circulating melanoma cells (48). Furthermore, EVs can orchestrate an immunosuppressive microenvironment: gastric cancer cell-derived EVs induce programmed death-ligand 1 (PD-L1) expression on neutrophils, while metastatic melanoma-derived EVs carry it directly on their surface, suppressing the function of CD8^+^ T cells. In this way, cancer cells can evade immune surveillance and grow (49, 50).

##### Tumor-Derived Extracellular Vesicles Promote Bone Metastasis by Inducing Bone Destruction

The engraftment of micrometastasis requires space for cancer cells from the primary tumor to settle and subsequently expand. EVs derived from different tumor types may contribute to preparing the pre-metastatic niche, providing the space necessary for circulating cancer cells to settle due to their osteoclastogenic properties, which are often associated with the ability to inhibit bone deposition by OBLs.

This behavior is evident in multiple myeloma-derived EVs (MM-EVs), which play a key role in altering the homeostasis between OBLs and OCLs. MM-EVs can directly induce OCL differentiation and activation. Raimondi et al. reported that exosomes from the MM cell lines U266 and MM1.S increase the viability and migration of Raw264.7 OCL progenitors. Exosomes derived from the MM cell line or the BM aspirates of MM patients can also stimulate Raw264.7 cells and primary OCL precursors from a healthy donor to give origin to multinucleate OCLs. OCL differentiation is confirmed by the increased gene and protein expression of tartrate-resistant acid phosphatase (TRAP) and MMP-9 (51).

A novel mechanism by which MM-EVs induce the unbalance between OCL and OBL activity is based on the involvement of hepatocyte growth factor (HGF). HGF-positive EVs have been found in the BM of a subset of MM patients and a subset of cultured MM primary cells (52), and recently *in vitro* studies reported the delivery of HGF to OBLs, where it triggers the activation of its receptor Met, resulting in the inhibition of the osteogenic activity (52) and increased secretion IL-11 (53). This cytokine, in turn, stimulates osteoclastogenesis (54, 55) and inhibits new bone deposition (54, 56). Of note, IL-11 is also reported to stimulate a RANKL-independent activation of osteoclastogenesis *via* the activation of the JAK1/STAT3 pathway and the consequent c-Myc expression (57).

MM-EVs have also been reported to alter bone homeostasis by inhibiting osteoblastic differentiation of MSC through the transfer of bioactive molecules. Li et al. showed that MM cell-derived exosome transfers the lncRNA RUNX2-AS1, which arises from the antisense strand of RUNX2 to MSCs decreasing their osteogenic potential (58). Moreover, MM-exosomes carry the growth factors amphiregulin (AREG), that affects bone homeostasis by triggering the epidermal growth factor receptor (EGFR) in MSCs. This results in inhibition of OBL differentiation, due to the increased release of the pro-osteoclastogenic cytokine IL-8 (59) and the decreased expression of the anti-osteoclastogenic OPG (59, 60). A similar effect is mediated by exosomes released from NSCLC, that, through their AREG cargo, activate EGFR phosphorylation which may induce the release of RANKL, the main osteoclastogenic factor (61).

Raimondo et al. also investigated the role of miRNA content of MM-exosome in osteoblastic differentiation (62). The analysis of miRNA content showed that the enrichment of Mir-1 29 hi5p in MM-EVs correlated with bone disease and disease progression from the premalignant form of smoldering myeloma to overt multiple myeloma. The effect of exosomal miR-129-5p in MSCs relies on its ability to inhibit the expression of the transcription factor Sp1 (63, 64) and the alkaline phosphatase (ALPL) required for OBL differentiation (65).

In breast cancer, recent *in vitro* studies reported the role of tumor derived EVs in promoting the formation of osteolytic lesions. Tiedemann et al. described the release of the cytosolic protein L-plastin from the breast cancer cell line MDA‐MB‐231-derived exosomes as a key event for OCL formation. According to the authors, L-plastin induces calcium oscillation and nuclear localization of the calcium-dependent nuclear factor of activated T-cells c1 (NFATc1), a key osteoclastogenic transcription factor (66), exploiting an L-plastin-based mechanism described also for other pro-osteoclastogenic factors produced by MDA-MB-231 cells (67). Interestingly, MDA-MB-231 cells deficient in L-plastin have a reduced capacity to stimulate osteolysis *in vivo* (66). Of note, MDA-MB-231 cells depleted of L-plastin and Peroxiredoxin 4 (PRDX4), another osteoclastogenic factor described also in prostate cancer cell PC3 (68), significantly reduced the osteolytic lesions in a murine model (66). Another study on MDA-MB-231 cells made it possible to identify another exosome-mediated mechanism based on miR-20a-5p, whose transfer induces primary murine BM pre-OCL differentiation. Osteoclastogenesis is confirmed by the upregulation of OCL-specific gene markers such as TRAP, calcitonin receptor, V-ATPase d2, and cathepsin K. The underlying molecular mechanisms involve the downregulation of a direct target of miR-20a-5p, the SRC kinase signaling inhibitor 1, SRCIN (69).

Additionally, breast cancer-derived exosomes inhibit OBL activity, as demonstrated by a recent *in vitro* study that reported that miR-218-5p inhibits the synthesis of collagen type I (which is essential for osseous matrix deposition), by directly downregulating COL1A1 expression in OBLs (70).

**Figure 1:**
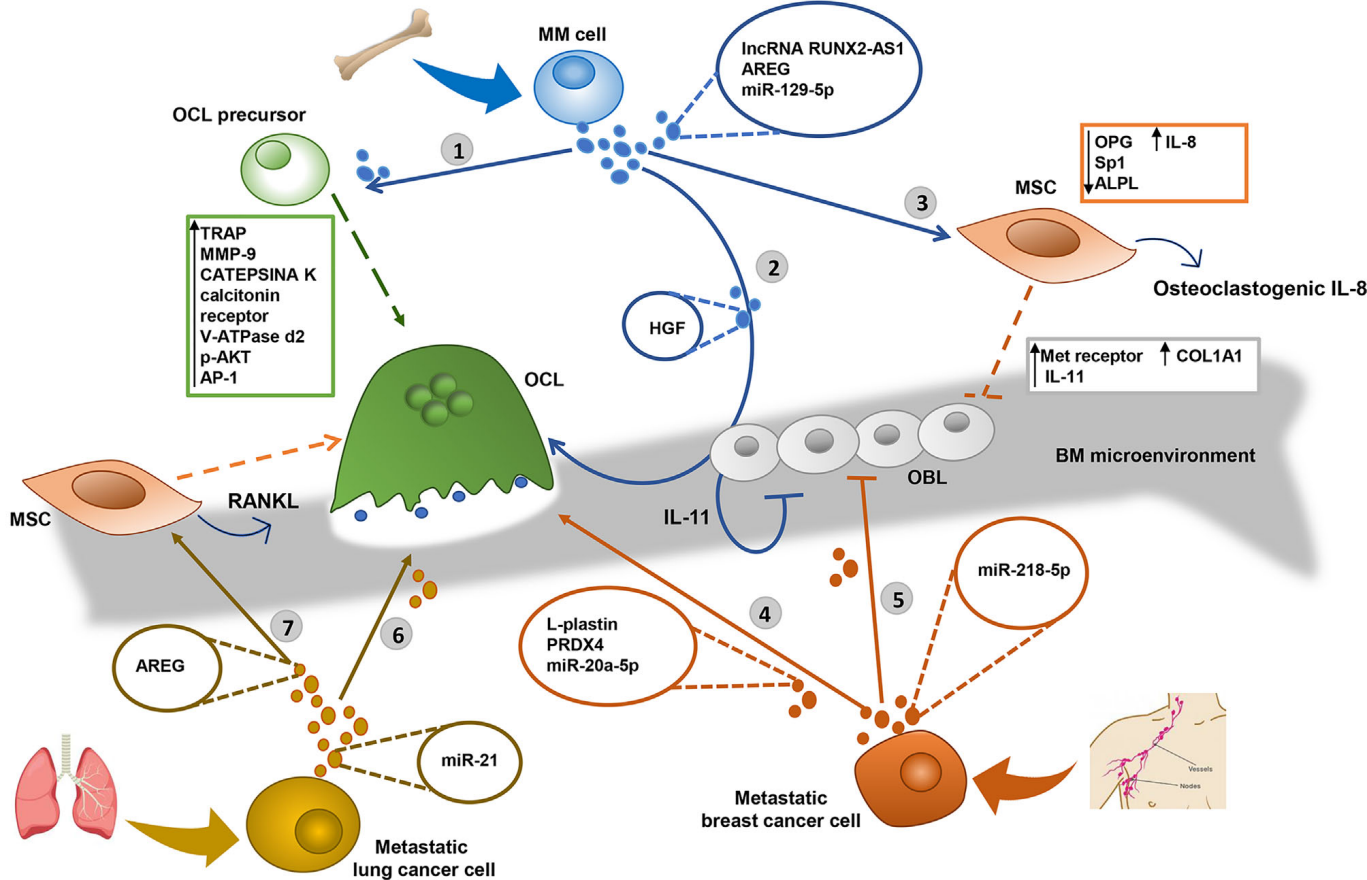
EVs derived from Multiple Myeloma (MM), breast and lung tumor metastatic cells alter the bone homeostasis inducing the osteolytic lesions. 1) MM derived-extracellular vesicles (MM-EVs) increase the migration of osteoclast (OCL) precursors and induce the OCL differentiation, producing tartrate-resistant acid phosphatase (TRAP) and MMP-9 expression (51). 2) MM-EVs inhibit osteoblast (OBL) activity through the transfer of the hepatocyte growth factor (HGF) by activating Met receptor activity (52) and induce osteoclastogenesis and the inhibition of new bone deposition through the secretion of IL-11 by OBL (53–56). 3) MM cells decrease the osteogenic potential of mesenchymal stromal cells (MSC) through the exosomal transfer of lncRNA RUNX2-AS1, which arises from the antisense strand of RUNX2, the transcription factor RUNT-related transcription factor 2 (58), and the growth factor amphiregulin (AREG), which induces the secretion of the pro-osteoclastogenic cytokine IL-8 (59) and downregulates the anti-osteoclastogenic osteoprotegerin (OPG) (59, 60). MM-EVs transfer miR-129-5p to recipient OBL and inhibit their differentiation through the downregulation of transcription factor Sp1 and the alkaline phosphatase ALPL (63, 64). 4) Metastatic breast cancer cells induce the OCL differentiation through the exosomal transfer of cytosolic protein L-plastin and Peroxiredoxin 4 (PRDX4) (66), and miR-20a-5p, inducing the upregulation of OCL-specific gene markers such as TRAP, calcitonin receptor, V-ATPase d2, and cathepsin K (69). 5) Breast cancer cells-derived EVs inhibit the OBL activity through the transfer of miR-218-5p which targets the COL1A1 expression reducing the collagen type I synthesis (70). 6) Metastatic lung cancer cells induce osteoclastogenesis through the exosomal transfer of miR-21 (71), which may activate the transcription factor AP-1 (72) and increases the expression of p-AKT (74). 7) Lung cancer cell derived EVs inhibit the osteogenic potential of MSC, through the transfer of AREG, inducing the release of the osteogenic receptor activator of nuclear factor k-B ligand (RANKL) (61).

The role of EVs has also been reported in bone metastatic lung cancer. Exosomal miR-21 released by the lung adenocarcinoma cell line A549 induces osteoclastogenesis by activating activator protein 1 (AP-1) (71). Indeed, miR-21 targets the tumor suppressor Pdcd4, which downregulates the transcription factor AP-1 (72) involved in RANKL-induced osteoclastogenesis (73). Moreover, miR-21 is reported to induce OCL differentiation by targeting tumor suppressor phosphatase and tensin homolog (PTEN), which induces apoptosis through the inhibition of the PI3K/AKT pathway. Indeed, treatment with a miR-21 mimic increases the expression of p-AKT in the murine monocyte cell line Raw264.7 cells (74), which regulates the GSK3β/NFATc1 signaling inducing OCL differentiation (75) (**Figure 1**).

##### Tumor-Derived Extracellular Vesicles Promote the Formation of Osteoblastic Bone Metastases

As osteoblastic bone lesions are particularly diffused in prostate cancer, it is not surprising that EVs are reported to play a key role in the bone lesions of advanced prostate cancer. *In vitro* and *in vivo* experiments indicate that exosomes isolated from the prostate cancer bone metastatic cell line, MDA PCa 2b, transfer miR-141-3p to OBLs. This micro-RNA downregulates the expression of its target gene DLC1 and activates the p38/MAPK signaling, which increases the expression of OPG and, consequently, unbalances OCL/OBL ratio in favor of the latter (76). Exosomes from another bone metastatic prostate cancer cell line, PC3, also displayed an inhibitory activity on osteoclastogenesis (77). A more recent *in vitro* study demonstrated that PC3 cell-derived EVs also enhance OBL viability by delivering several miRNAs and mRNAs. In turn, “educated” OBLs support prostate cancer cell growth in a co-culture system (47). The miRNA 21 was identified as the most abundant miRNA in EVs from prostate cancer cells involved in the viability of OBLs. Interestingly, prostate cancer-derived EVs transfer CSF-1, VEGFA, MCP1, RUNX2, and FGF2 transcripts within recipient OBLs, but only CSF-1 content is significantly increased in the recipient cells, suggesting a mechanism controlling the selective use of EVs-cargo molecules in recipient OBLs.

**Figure 2:**
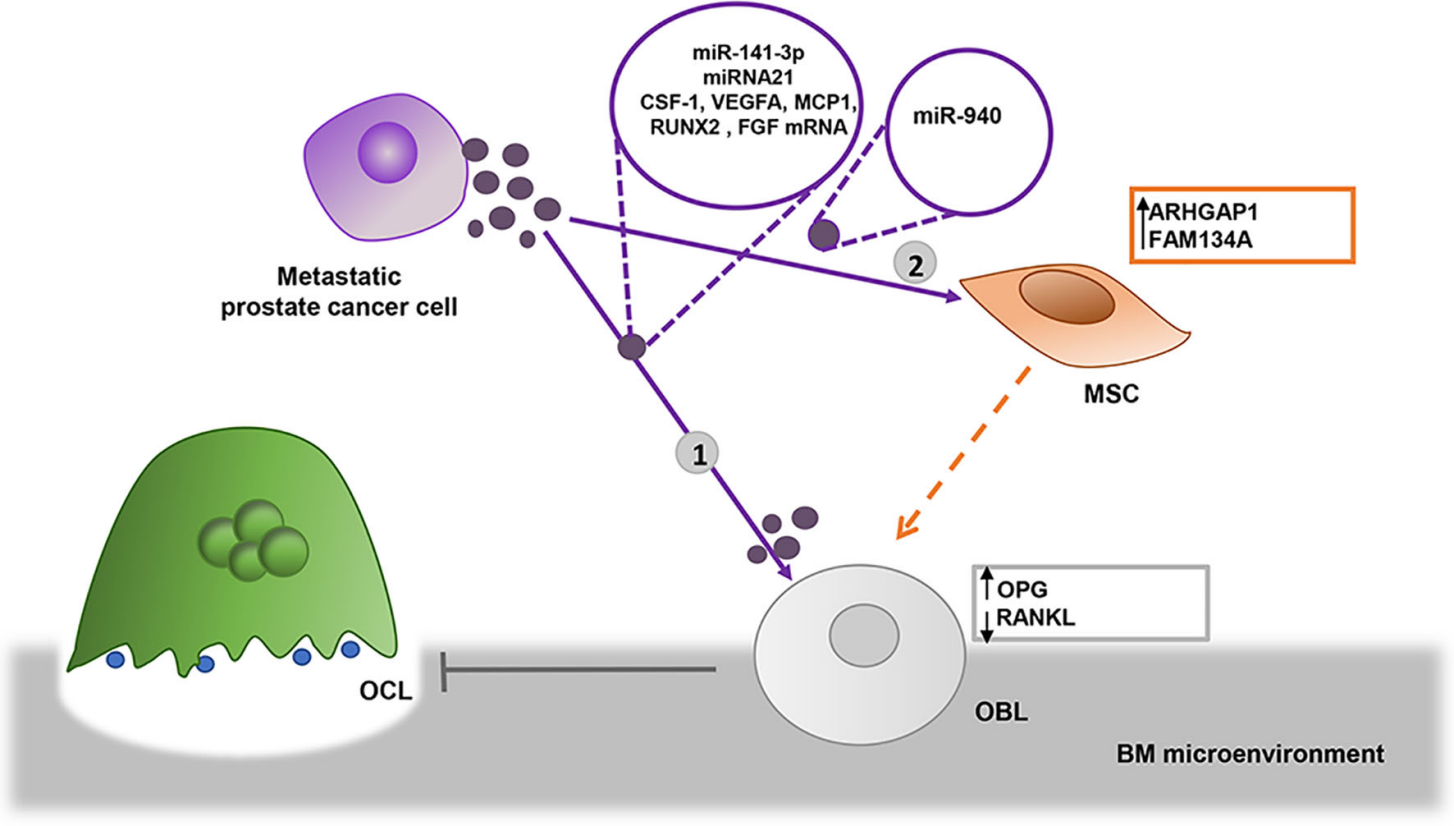
EVs derived from prostate metastatic cells induce osteoblastic lesions. 1) Bone metastatic prostate cancer cells transfer miR-141-3p into osteoblasts (OBL) *via* EVs, where it downregulates the expression of osteoprotegerin (OPG), altering the balance between OBL/OCL ratio, promoting OBL activity as a result (76). EVs from prostate cells enhance OBL viability by delivering miRNA 21 and CSF-1, VEGFA, MCP1, RUNX2, and FGF2 transcripts (47) 2) Prostate cancer cells may indirectly favor osteoblastic bone lesions transferring miR-940 *via* EVs, which target Rho GTPase Activating Protein 1 (ARHGAP1) and Reticulophagy regulator 2, Family with sequence similarity 134, member A (FAM134A) in mesenchymal stromal cells (MSCs) induce their differentiation in OBLs (44).

Another mechanism was recently reported, by which EVs derived from prostate cancer cells may favor osteoblastic bone lesions. They carry miR-940 which targets Rho GTPase Activating Protein 1 (ARHGAP1), Reticulophagy regulator 2, Family with sequence similarity 134 (FAM134A), member A, in mesenchymal cells and induces their differentiation in OBLs (44). ARHGAP1 is involved in the osteogenic differentiation of MSCs through the RhoA/ROCK pathway regulation (78); whereas the role of FAM134A is unclear (**Figure 2**).

##### Bone Cell Populations May Release EVs to Support Metastatic Tumor Cell Growth

Once “educated”, cells of the bone microenvironment may support bone metastasis formation through exosome release. Here, we will report evidence involving the role of mesenchymal cells, including BM MSCs, OBLs, and OCLs.

The role of BM-MSCs derived EVs (MSC-EVs) in promoting bone metastases is extensively documented in MM. MSC-EVs create a supportive microenvironment for MM progression and drug resistance (79, 80). Roccaro et al. showed that the exosomes isolated from the BM-MSCs (MSC-exosomes) of MM patients induce MM cell proliferation *in vitro* and promote the dissemination and metastasis to different skeletal sites *in vivo*. This behavior is not observed with exosomes from the primary BM-MSCs of healthy donors (81). This evidence suggests that BM-MSC in the tumor microenvironment are educated in supporting bone colonization. The mechanism at the base of the MSC-exosome effect may rely on exosome content.

MM MSC-exosomes are characterized by higher levels of miRNAs and oncogenic proteins compared to MSC-exosomes from healthy subjects (81) and display other relevant differences. For instance, MM MSC-exosomes have an increased content of IL-6, CCL2, junction plakoglobin, or fibronectin (81), already known to be involved in MM progression (82, 83). Additionally, MM MSC-exosomes are characterized by a lower level of miR-15a; this microRNA is associated with suppression of tumor proliferation (81). Consistently, MM MSC-exosomes deficient for miR-15a promotes MM cell dissemination to different skeletal sites *in vivo* (81).

According to Ho et al., MSC-exosome quantity and quality are positively regulated by the histone deacetylase 3 (HDAC3), which is overexpressed in MM MSCs compared to the MSCs of a healthy population (84). The inhibition of HDAC3 gene and protein expression *in vitro* and *in vivo* affected not only the proliferation of MM cells co-cultured with MSCs but also the production of MSC-exosomes. Inhibitory experiments demonstrated that HDAC3 plays its regulative role in MSCs by stimulating the expression of a specific ESCRT complex component, Tumor Susceptibility Gene 101 (TSG101), and increasing the MSC-exosome content of miR380 and miR382 (84). miR382 is overexpressed in MM patients and targets tumor suppressor genes involved in tumor proliferation and survival (85), while miR380 is reported to attenuate p53 signaling in neuroblastoma (86).

Although the role of OBL- and OCL-derived EVs in bone metastasis is less investigated, several pieces of evidence suggest that they contribute to altering the bone homeostasis, possibly also activating positive feedback in osteoblastic or osteolytic metastatic lesions. In particular, OBL-derived exosomes carry different mRNAs and proteins depending on the OBL differentiation stage, i.e., immature proliferative cells versus mature mineralizing OBLs (87–89). Mineralizing OBLs can enhance osteogenesis, transferring exosomes that can regulate microRNA expression in BM MSCs, and thereby promoting their differentiation into OBLs through the activation of the WNT, insulin, and TGF‐β signaling pathways, as well as the mineralizing activity by activating the calcium signaling pathway (90). Interestingly, a recent study suggests that OBL increase in the context of bone osteoblastic lesions might favor tumor growth *via* a pro-tumor communication mediated by EVs. The exosomes (87) and EVs released by mineralizing OBLs induce PC3 prostate cancer cell growth *in vitro* (89).

As expected, OCL-derived EVs play the opposite role and could amplify the bone destructive effect of osteolytic metastatic lesions. In particular, an *in vitro* and *in vivo* study reported that OCLs secrete miR-214-enriched exosomes that specifically target OBLs through the interaction between ephrinA2 and ephrin type-A receptor 2 (EphA2). The OCL exosome-mediated transfer of miR-214 into OBLs finally results in the inhibition of OBL activity.

#### Potential of EV-Based Anti-Metastatic Therapeutic Approaches

The possible therapeutic role of EVs has been recently recognized in a position paper by the International Society of extracellular vesicles (91). The crucial role played by EVs in the pathological communication between cancer and bone cell populations during bone metastasis formation suggests that EVs and their content could be a target of novel therapeutic approaches to restore bone homeostasis to prevent the formation of widespread metastasis.

Since the EVs secreted by tumor cells are pivotal players in the metastasis process, several strategies were developed to neutralize the effects. These include impairing biogenesis and the release of EVs by cancer cells, inhibiting their uptake by recipient cells, and removing them from circulation. Recently the inhibition of EVs release has been reported as a promising approach in the different types of tumors that develop bone metastasis, including breast, lung, and liver cancer, as well as MM.

Administration of sulfisoxazole (SFX), an FDA approved antibiotic, inhibits the biogenesis of EVs from breast cancer cells and prevents EV secretion *via* downregulation of the components of the ESCRT-dependent machine, such as CD63, RAB27, and RAB7 (92). SFX administration results in the suppression of breast cancer cell growth and metastasis. Although the work was focused on lung and liver metastasis, it is reasonably conceivable that a similar suppressive effect may be extended also to bone metastasis (92). The authors clarified the underlying molecular mechanism by demonstrating that SFX targeted endothelin receptor A associated with EV secretion by breast cancer cells, thereby reducing the number of released EVs and their protein cargo (92). The key role of RAB proteins in EV secretion was also reported by Bobrie et al., who demonstrated that RAB27b knockdown interfered with EV secretion and resulted in decreased tumor growth and lung metastasis of 4T1 breast cancer cells *in vivo* (93).

EV secretion blockage was also obtained through the use of the sphingomyelinase inhibitor GW4869, which specifically inhibits exosome secretion (94). Fabbri et al. observed that mice treated with GW4869 formed a significantly lower number of lung metastasis when injected with lung cancer cells LLC. The inhibitory effect could be rescued if GW4869-bearing mice were injected with EVs derived from LLC cells (95). Faict et al. showed that blocking EVs secretion with GW4869 in an *in vivo* model of MM interfered with osteolysis and augmented cortical bone volume (96), demonstrating that GW4869 may restore the bone homeostasis altered by metastatic cancer cells. Interestingly, GW4869 displays a further important outcome since it diminishes the resistance to bortezomib eliciting a strong anti-tumor response when administered in combination with this drug (96). A similar effect on chemoresistance was also reported in doxorubicin (Dox) resistant tumors, one of the challenges of cancer chemotherapy. Khan et al. demonstrated that Dox resistance can be overcome by blocking EV secretion with ketotifen (97). Ketotifen’s ability to sensitize different cell tumor lines – including HeLa (cervix carcinoma cells), MCF7, and BT549 (breast cancer cells) - to Dox was proportional to its effect on exosomes release (97).

Another way to neutralize the EV-mediated promotion of metastasis is to remove them from circulation. In xenograft breast cancer model mice, circulating EVs derived from tumor cells were captured by anti-human CD9 and anti-human CD63 antibodies stimulating EV removal by macrophages internalization, associated with a decreased metastasis formation into the lungs, lymph nodes, and thoracic cavity (98). Neutralization of EVs circulation by monoclonal antibodies has been proposed for a primary bone cancer such as osteosarcoma. Baglio et al. provided evidence that osteosarcoma-derived EV are enriched with a membrane-bound form of TGFβ, which induces human MSCs to produce IL6 stimulating osteosarcoma growth and lung metastasis formation in an orthotopic xenograft model (99). The authors demonstrated that the inhibition of IL6R signaling by tocilizumab prevented osteosarcoma metastasis formation and suggested that TGFβ-blocking agents could be tested as new therapeutic options for osteosarcoma patients (99).

Blocking EV uptake can also interfere with the preparation of a pre-metastatic niche by EVs. Hoshino et al. showed that targeting the distinct integrins on EVs by integrin-blocking decoy peptides ablated lung and liver-metastatic EV uptake in an integrin-specific and organ-specific manner, reducing the formation of metastasis in these organs (100).

The ability of EVs to efficiently transport molecules into targeted cells makes them optimal drug delivery carriers. Different from artificial nanoparticles, EVs can overcome limitations such as toxicity and low half-life. Syngenic EVs cannot be rejected by the immune system and their protein cargo is protected, thus conferring it a longer half-life. Moreover, the organ tropism of EVs makes them suitable vehicles for transporting drugs and nucleic acids to specific metastatic sites (100).

EV-based strategies use both unmodified and engineered EVs loaded with different molecules and compounds.

Naïve EVs derived from MSCs can be used in the tissue repair of skeletal lesions arising from osteolytic metastasis. The role of BM mesenchymal stem cells in repairing damaged organs and tissue is well documented (101). Recent evidence reports that EVs produced by these cells display great potential in bone regeneration due to EV content enriched with several signaling molecules that can promote tissue bone repair. Qin et al. demonstrated that the EVs derived from BM mesenchymal stem cells could be uptaken by OBLs and deliver osteogenic miR-196a regulating osteogenic gene expression and osteoblastic differentiation (102). The same type of EVs stimulated bone regeneration in Sprague Dawley rats with calvarial defects (102) as well as osteogenic differentiation through the activation of the PI3/AKT pathway, which promoted bone formation and neovascularization indicating potential in bone repair (103).

Several therapeutic applications have been proposed for EVs loaded with therapeutic molecules or siRNAs/miRNAs or for EVs modified to improve their efficacy.

Some chemotherapeutic agents, such as Dox and paclitaxel (PTX), have been delivered through EVs to target tumor cells. Tian et al. obtained engineered EVs derived from mouse immature dendritic cells, expressing the exosomal membrane protein Lamp2b fused to the αv integrin-specific iRGD peptide (104). These EVs, loaded with Dox and injected in a breast tumor mouse model, were able to specifically deliver the drug leading to the inhibition of tumor growth without overt toxicity (104).

Another study revealed that MSCs exposed to 2000 ng/ml PTX were able to package and deliver the active drug into EVs that showed a strong anti-proliferative activity on the human pancreatic cell line CFPAC-1 (105). The group of Batrakova showed that both naïve EVs from macrophage loaded directly with PTX or EVs modified with PEG-AA vector moiety to improve their circulation and loaded with PTX could accumulate in cancer cells and target and reduce pulmonary metastases in a model of murine Lewis lung carcinoma (106, 107).

EVs loaded with PTX are used also in combination with other agents. Systemic administration of EV formulations with oncolytic virus in combination with PTX improved tumor-selective delivery, peritumoral immune-response associated with the targeted delivery of the virus, enhanced immunogenicity and infiltration of CD4+ and CD8+ T-cells in a lung cancer mouse model, indicating the possible application of these EVs in treating primary and metastatic cancers (108). The effect of EVs isolated from proliferating MSCs after taxol treatment was studied on different tumors *in vitro* and *in vivo* (109). EVs loaded with taxol exhibited cancer cell cytotoxicity similarly to the administration of free taxol in three different tumor cell types including lung, ovarian, and breast cancer. The tumor therapeutic effect of the EVs in a mouse breast cancer model displayed a significant reduction of tumor size and metastasis formation in lungs, liver, spleen but not in BM (109). Reports concerning the potential of EVs as a carrier for anti-tumor agents suggest that this is a promising approach that needs to be coupled with a specific delivery in the BM microenvironment. Thus, EVs from BM mesenchymal stem cells were used as carriers to load Dox for osteosarcoma treatment (110). These Dox-loaded EVs exhibited a high level of uptake and displayed strong antitumor effects on osteosarcoma cells while no cytotoxicity for myocardial cell lines was observed. Interestingly, a recent report suggests that EVs can be delivered to the specific cellular targets relevant for bone metastasis formation. Specifically, OBL-derived EVs can be used for carrying pharmacological molecules including anti‐osteoclastic agents, such as dasatinib and zoledronate, which are very effective in inhibiting OCL activity *in vitro* and *in vivo* (111).

The ability of EVs to carry small RNAs, such as siRNA and miRNA, was exploited in therapeutic approaches to counteract tumor growth and metastasis formation. Techniques to encapsulate siRNAs and miRNAs were developed to ensure successful delivery to receiving cells (112). The group of Wood engineered dendritic cells to express Lamp2b, an exosomal membrane protein, which fused to the neuron-specific RVG peptide. To verify the specificity of delivery, GAPDH siRNA was loaded on these EVs, which were injected into mice and specifically delivered to neurons, microglia, and oligodendrocytes in the brain, resulting in the specific knockdown of GAPDH gene expression (113). Another example of the successful delivery of exosomal siRNA was reported by Wang et al., which showed that EVs loaded with transient receptor potential polycystic 2 (TRPP2) siRNA were able to reduce epithelial mesenchymal transition by suppressing TRPP2 expression in a cell line of human pharyngeal squamous cell carcinoma (114).

Since miRNAs play a documented role in bone metastasis (115), EVs carrying miRNAs might be used not only as biomarkers but for therapeutic purposes. The delivery of exosomal miRNAs into tumor cells was demonstrated by Katakowski et al. They found that EVs derived from miR-146b-expressing BM MSCs and injected in a rat model of primary brain tumor, significantly reduced glioma xenograft growth (116).

Few studies have described the treatment of bone metastasis using EVs loaded with miRNAs. Research by Valencia et al. indicated that the bone colonization and osteolytic lesions induced by lung cancer can be deranged *in vivo* by preconditioning the bone environment with EVs, isolated from the human metastatic non-small cell lung cancer cell line A549 overexpressing miR-192. Micro-RNA 192-enriched EVs reduce the expression of key angiogenic factors such as IL-8, ICAM1, and CXCL1 in endothelial precursor cells, preventing tumor‐induced angiogenic switch and, consequently, reducing bone colonization and metastatic burden (117). Another miRNA, let-7, was shown to be involved in supporting OBL function. The let-7 enriched exosomes produced by OBLs enhance osteogenesis by regulating high‐mobility group AT‐hook 2 (HMGA2) and AXIN2 (118), suggesting that an approach based on Let‐7 loaded vesicles could be helpful to inhibit osteolytic bone lesions. Khani et al. demonstrated that an *in vivo* treatment with tumor EVs loaded with exogenous Let-7 could increase the survival rate of breast cancer-bearing mice and induced a reduction in tumor growth (119). Moreover, Ohno et al. reported that the EVs produced by cells engineered to express the transmembrane domain of the PDGF receptor fused to the GE11 peptide that binds specifically to EGFR were able to efficiently deliver let-7a to EGFR-expressing breast cancer cells. GE11-positive exosomes containing let-7 were demonstrated to inhibit tumor development *in vivo* (120).

An approach that uses natural EVs to deliver specific anti-tumor molecules should also take into account the necessity of reducing or avoiding the delivery of pro-tumor molecules, especially when EVs are produced by cancer cells. For instance, Hong and colleagues (121) set up a cell-free tumor vaccine using tumor-derived exosomes carrying tumor associated antigens but had to deplete the exosomes of TGF-β1 to avoid the immunosuppressive effect. This issue should be considered given the possible use of EVs to inhibit bone metastasis. Indeed, TGF-β1 is a key cytokine in osteolytic bone lesions and its presence in breast cancer-derived EVs has been shown to increase the accumulation and function of immature myeloid cells and boost osteoclastic bone resorption (122).

In conclusion, emerging evidence shows that counteracting the function of tumor-derived EVs in the metastatic process may be a novel successful strategy. Further research is needed to investigate the function and modalities of therapeutic EV-based applications including research targeting bone metastasis, which may involve either the inhibition of EV secretion and the uptake by recipient BM cells or their circulation, as well as the appropriate modification of EV cargo with molecules capable of interfering with bone metastasis.

## Author Contributions

DG, VC, RC and NP collected the relevant literature and drafted the manuscript with input from all authors. EL and MB searched literature and provided advice. RC and NP provided critical feedback and revised the manuscript. All authors contributed to the article and approved the submitted version.

## Funding

This research was funded by Associazione Italiana Ricerca sul Cancro, AIRC Investigator Grant (grant number 20614) and by the University of Milano (grant number Linea 2B-2019- Dept. Health Sciences) to RC, and an AIRC post-doctoral fellowship to VC (financed with Investigator Grant 20614 to RC). Università degli Studi di Milano awarded DG with a PhD fellowship in Experimental Medicine.

## Conflict of Interest

The authors declare that the research was conducted in the absence of any commercial or financial relationships that could be construed as a potential conflict of interest.

## References

[1] Russell AE Sneider A Witwer KW Bergese P Bhattacharyya SN Cocks A. Biological membranes in EV biogenesis, stability, uptake, and cargo transfer: an ISEV position paper arising from the ISEV membranes and EVs workshop. J Extracellular Vesicles (2019) 8(1):1684862. 10.1080/20013078.2019.1684862 31762963 PMC6853251

[2] van Niel G D’Angelo G Raposo G. Shedding light on the cell biology of extracellular vesicles. Nat Rev Mol Cell Biol (2018) 19(4):213–28. 10.1038/nrm.2017.125 29339798

[3] Huotari J Helenius A. Endosome maturation. EMBO J (2011) 30(17):3481–500. 10.1038/emboj.2011.286 PMC3181477 21878991

[4] Hessvik NP Llorente A. Current knowledge on exosome biogenesis and release. Cell Mol Life Sci CMLS (2018) 75(2):193–208. 10.1007/s00018-017-2595-9 28733901 PMC5756260

[5] Hurwitz SN Conlon MM Rider MA Brownstein NC Meckes DG Jr. Nanoparticle analysis sheds budding insights into genetic drivers of extracellular vesicle biogenesis. J Extracellular Vesicles (2016) 5:31295. 10.3402/jev.v5.31295 27421995 PMC4947197

[6] van Niel G Charrin S Simoes S Romao M Rochin L Saftig P. The tetraspanin CD63 regulates ESCRT-independent and -dependent endosomal sorting during melanogenesis. Dev Cell (2011) 21(4):708–21. 10.1016/j.devcel.2011.08.019 PMC3199340 21962903

[7] Trajkovic K Hsu C Chiantia S Rajendran L Wenzel D Wieland F. Ceramide triggers budding of exosome vesicles into multivesicular endosomes. Sci (New York NY) (2008) 319(5867):1244–7. 10.1126/science.1153124 18309083

[8] Pfeffer SR. Unsolved mysteries in membrane traffic. Annu Rev Biochem (2007) 76:629–45. 10.1146/annurev.biochem.76.061705.130002 17263661

[9] Tricarico C Clancy J D’Souza-Schorey C. Biology and biogenesis of shed microvesicles. Small GTPases (2017) 8(4):220–32. 10.1080/21541248.2016.1215283 PMC5680703 27494381

[10] Muralidharan-Chari V Clancy J Plou C Romao M Chavrier P Raposo G. ARF6-regulated shedding of tumor cell-derived plasma membrane microvesicles. Curr Biol CB (2009) 19(22):1875–85. 10.1016/j.cub.2009.09.059 PMC3150487 19896381

[11] Mulcahy LA Pink RC Carter DR. Routes and mechanisms of extracellular vesicle uptake. J Extracellular Vesicles (2014) 3(1):24641. 10.3402/jev.v3.24641 PMC4122821 25143819

[12] Jurj A Zanoaga O Braicu C Lazar V Tomuleasa C Irimie A. A Comprehensive Picture of Extracellular Vesicles and Their Contents. Molecular Transfer to Cancer Cells. Cancers (2020) 12(2):298. 10.3390/cancers12020298 PMC7072213 32012717

[13] Fornetti J Welm AL Stewart SA. Understanding the Bone in Cancer Metastasis. J Bone Mineral Res Off J Am Soc Bone Mineral Res (2018) 33(12):2099–113. 10.1002/jbmr.3618 30476357

[14] Coleman RE. Clinical features of metastatic bone disease and risk of skeletal morbidity. Clin Cancer Res an Off J Am Assoc Cancer Res (2006) 12(20 Pt 2):6243s–49s. 10.1158/1078-0432.CCR-06-0931 17062708

[15] Rizzoli R Body JJ Brandi ML Cannata-Andia J Chappard D El Maghraoui A. Cancer-associated bone disease. Osteoporosis Int J stablished as result cooperation between Eur Foundation Osteoporosis Natl Osteoporosis Foundation USA (2013) 24(12):2929–53. 10.1007/s00198-013-2530-3 PMC5104551 24146095

[16] Hiraga T. Bone metastasis: Interaction between cancer cells and bone microenvironment. J Oral Biosci (2019) 61(2):95–8. 10.1016/j.job.2019.02.002 31109867

[17] Weidle UH Birzele F Kollmorgen G Ruger R. Molecular Mechanisms of Bone Metastasis. Cancer Genomics Proteomics (2016) 13(1):1–12. 26708594

[18] Coleman RE. Metastatic bone disease: clinical features, pathophysiology and treatment strategies. Cancer Treat Rev (2001) 27(3):165–76. 10.1053/ctrv.2000.0210 11417967

[19] Palumbo A Anderson K. Multiple myeloma. New Engl J Med (2011) 364(11):1046–60. 10.1056/NEJMra1011442 21410373

[20] Ren G Esposito M Kang Y. Bone metastasis and the metastatic niche. J Mol Med (2015) 93(11):1203–12. 10.1007/s00109-015-1329-4 PMC4636917 26275789

[21] Kenkre JS Bassett J. The bone remodelling cycle. Ann Clin Biochem (2018) 55(3):308–27. 10.1177/0004563218759371 29368538

[22] Zhao C Irie N Takada Y Shimoda K Miyamoto T Nishiwaki T. Bidirectional ephrinB2-EphB4 signaling controls bone homeostasis. Cell Metab (2006) 4(2):111–21. 10.1016/j.cmet.2006.05.012 16890539

[23] Cheng C Ji Z Sheng Y Wang J Sun Y Zhao H. Aphthous ulcer drug inhibits prostate tumor metastasis by targeting IKKvarepsilon/TBK1/NF-kappaB signaling. Theranostics (2018) 8(17):4633–48. 10.7150/thno.26687 PMC6160770 30279728

[24] Kusumbe AP Ramasamy SK Adams RH. Coupling of angiogenesis and osteogenesis by a specific vessel subtype in bone. Nature (2014) 507(7492):323–8. 10.1038/nature13145 PMC4943525 24646994

[25] Xie H Cui Z Wang L Xia Z Hu Y Xian L. PDGF-BB secreted by preosteoclasts induces angiogenesis during coupling with osteogenesis. Nat Med (2014) 20(11):1270–8. 10.1038/nm.3668 PMC4224644 25282358

[26] Dar HY Azam Z Anupam R Mondal RK Srivastava RK. Osteoimmunology: The Nexus between bone and immune system. Front Biosci (2018) 23:464–92. 10.2741/4600 28930556

[27] Mirosavljevic D Quinn JM Elliott J Horwood NJ Martin TJ. Gillespie MT. T-cells mediate an inhibitory effect of interleukin-4 on osteoclastogenesis. J Bone Mineral Res Off J Am Soc Bone Mineral Res (2003) 18(6):984–93. 10.1359/jbmr.2003.18.6.984 12817750

[28] Zhu X Zeng Z Qiu D Chen J. Vgamma9Vdelta2 T cells inhibit immature dendritic cell transdifferentiation into osteoclasts through downregulation of RANK, cFos and ATP6V0D2. Int J Mol Med (2018) 42(4):2071–9. 10.3892/ijmm.2018.3791 PMC6108864 30066839

[29] Horwood NJ Kartsogiannis V Quinn JM Romas E Martin TJ Gillespie MT. Activated T lymphocytes support osteoclast formation in vitro. Biochem Biophys Res Commun (1999) 265(1):144–50. 10.1006/bbrc.1999.1623 10548505

[30] Kotake S Udagawa N Hakoda M Mogi M Yano K Tsuda E. Activated human T cells directly induce osteoclastogenesis from human monocytes: possible role of T cells in bone destruction in rheumatoid arthritis patients. Arthritis Rheum (2001) 44(5):1003–12. 10.1002/1529-0131(200105)44:5<1003::AID-ANR179>3.0.CO;2-# 11352231

[31] Colucci S Brunetti G Rizzi R Zonno A Mori G Colaianni G. T cells support osteoclastogenesis in an in vitro model derived from human multiple myeloma bone disease: the role of the OPG/TRAIL interaction. Blood (2004) 104(12):3722–30. 10.1182/blood-2004-02-0474 15308561

[32] Ullah TR. The role of CXCR4 in multiple myeloma: Cells’ journey from bone marrow to beyond. J Bone Oncol (2019) 17:100253. 10.1016/j.jbo.2019.100253 31372333 PMC6658931

[33] Mirandola L Apicella L Colombo M Yu Y Berta DG Platonova N. Anti-Notch treatment prevents multiple myeloma cells localization to the bone marrow via the chemokine system CXCR4/SDF-1. Leukemia (2013) 27(7):1558–66. 10.1038/leu.2013.27 23354012

[34] Smith MC Luker KE Garbow JR Prior JL Jackson E Piwnica-Worms D. CXCR4 regulates growth of both primary and metastatic breast cancer. Cancer Res (2004) 64(23):8604–12. 10.1158/0008-5472.CAN-04-1844 15574767

[35] Weilbaecher KN Guise TA McCauley LK. Cancer to bone: a fatal attraction. Nat Rev Cancer (2011) 11(6):411–25. 10.1038/nrc3055 PMC3666847 21593787

[36] Mundy GR. Metastasis to bone: causes, consequences and therapeutic opportunities. Nat Rev Cancer (2002) 2(8):584–93. 10.1038/nrc867 12154351

[37] Colombo M Thummler K Mirandola L Garavelli S Todoerti K Apicella L. Notch signaling drives multiple myeloma induced osteoclastogenesis. Oncotarget (2014) 5(21):10393–406. 10.18632/oncotarget.2084 PMC4279381 25257302

[38] Zacharia B Subramaniam D Joy J. Skeletal Metastasis-an Epidemiological Study. Indian J Surg Oncol (2018) 9(1):46–51. 10.1007/s13193-017-0706-6 29563734 PMC5856695

[39] Colombo M Giannandrea D Lesma E Basile A Chiaramonte R. Extracellular Vesicles Enhance Multiple Myeloma Metastatic Dissemination. Int J Mol Sci (2019) 20(13):3236. 10.3390/ijms20133236 PMC6650870 31266187

[40] Grange C Tapparo M Collino F Vitillo L Damasco C Deregibus MC. Microvesicles released from human renal cancer stem cells stimulate angiogenesis and formation of lung premetastatic niche. Cancer Res (2011) 71(15):5346–56. 10.1158/0008-5472.CAN-11-0241 21670082

[41] Di Modica M Regondi V Sandri M Iorio MV Zanetti A Tagliabue E. Breast cancer-secreted miR-939 downregulates VE-cadherin and destroys the barrier function of endothelial monolayers. Cancer Lett (2017) 384:94–100. 10.1016/j.canlet.2016.09.013 27693459

[42] Liang H Yan X Pan Y Wang Y Wang N Li L. MicroRNA-223 delivered by platelet-derived microvesicles promotes lung cancer cell invasion via targeting tumor suppressor EPB41L3. Mol Cancer (2015) 14:58. 10.1186/s12943-015-0327-z 25881295 PMC4360939

[43] Mannavola F Tucci M Felici C Passarelli A D’Oronzo S Silvestris F. Tumor-derived exosomes promote the in vitro osteotropism of melanoma cells by activating the SDF-1/CXCR4/CXCR7 axis. J Trans Med (2019) 17(1):230. 10.1186/s12967-019-1982-4 PMC6642540 31324252

[44] Hashimoto K Ochi H Sunamura S Kosaka N Mabuchi Y Fukuda T. Cancer-secreted hsa-miR-940 induces an osteoblastic phenotype in the bone metastatic microenvironment via targeting ARHGAP1 and FAM134A. Proc Natl Acad Sci U States America (2018) 115(9):2204–9. 10.1073/pnas.1717363115 PMC5834702 29440427

[45] O’Brien K Rani S Corcoran C Wallace R Hughes L Friel AM. Exosomes from triple-negative breast cancer cells can transfer phenotypic traits representing their cells of origin to secondary cells. Eur J Cancer (2013) 49(8):1845–59. 10.1016/j.ejca.2013.01.017 23453937

[46] Dai J Escara-Wilke J Keller JM Jung Y Taichman RS Pienta KJ. Primary prostate cancer educates bone stroma through exosomal pyruvate kinase M2 to promote bone metastasis. J Exp Med (2019) 216(12):2883–99. 10.1084/jem.20190158 PMC6888980 31548301

[47] Probert C Dottorini T Speakman A Hunt S Nafee T Fazeli A. Communication of prostate cancer cells with bone cells via extracellular vesicle RNA; a potential mechanism of metastasis. Oncogene (2019) 38(10):1751–63. 10.1038/s41388-018-0540-5 PMC6372071 30353168

[48] Peinado H Aleckovic M Lavotshkin S Matei I Costa-Silva B Moreno-Bueno G. Melanoma exosomes educate bone marrow progenitor cells toward a pro-metastatic phenotype through MET. Nat Med (2012) 18(6):883–91. 10.1038/nm.2753 PMC3645291 22635005

[49] Shi Y Zhang J Mao Z Jiang H Liu W Shi H. Extracellular Vesicles From Gastric Cancer Cells Induce PD-L1 Expression on Neutrophils to Suppress T-Cell Immunity. Front Oncol (2020) 10:629. 10.3389/fonc.2020.00629 32477934 PMC7237746

[50] Chen G Huang AC Zhang W Zhang G Wu M Xu W. Exosomal PD-L1 contributes to immunosuppression and is associated with anti-PD-1 response. Nature (2018) 560(7718):382–6. 10.1038/s41586-018-0392-8 PMC6095740 30089911

[51] Raimondi L De Luca A Amodio N Manno M Raccosta S Taverna S. Involvement of multiple myeloma cell-derived exosomes in osteoclast differentiation. Oncotarget (2015) 6(15):13772–89. 10.18632/oncotarget.3830 PMC4537049 25944696

[52] Stromme O Psonka-Antonczyk KM Stokke BT Sundan A Arum CJ Brede G. Myeloma-derived extracellular vesicles mediate HGF/c-Met signaling in osteoblast-like cells. Exp Cell Res (2019) 383(1):111490. 10.1016/j.yexcr.2019.07.003 31283912

[53] Hjertner O Torgersen ML Seidel C Hjorth-Hansen H Waage A Borset M. Hepatocyte growth factor (HGF) induces interleukin-11 secretion from osteoblasts: a possible role for HGF in myeloma-associated osteolytic bone disease. Blood (1999) 94(11):3883–8. 10572104

[54] Girasole G Passeri G Jilka RL Manolagas SC. Interleukin-11: a new cytokine critical for osteoclast development. J Clin Invest (1994) 93(4):1516–24. 10.1172/JCI117130 PMC294166 8163655

[55] McCoy EM Hong H Pruitt HC Feng X. IL-11 produced by breast cancer cells augments osteoclastogenesis by sustaining the pool of osteoclast progenitor cells. BMC Cancer (2013) 13:16. 10.1186/1471-2407-13-16 23311882 PMC3554506

[56] Hughes FJ Howells GL. Interleukin-11 inhibits bone formation in vitro. Calcif Tissue Int (1993) 53(5):362–4. 10.1007/BF01351844 8287326

[57] Liang M Ma Q Ding N Luo F Bai Y Kang F. IL-11 is essential in promoting osteolysis in breast cancer bone metastasis via RANKL-independent activation of osteoclastogenesis. Cell Death Dis (2019) 10(5):353. 10.1038/s41419-019-1594-1 31040267 PMC6491651

[58] Li B Xu H Han H Song S Zhang X Ouyang L. Exosome-mediated transfer of lncRUNX2-AS1 from multiple myeloma cells to MSCs contributes to osteogenesis. Oncogene (2018) 37(41):5508–19. 10.1038/s41388-018-0359-0 29895968

[59] Raimondo S Saieva L Vicario E Pucci M Toscani D Manno M. Multiple myeloma-derived exosomes are enriched of amphiregulin (AREG) and activate the epidermal growth factor pathway in the bone microenvironment leading to osteoclastogenesis. J Hematol Oncol (2019) 12(1):2. 10.1186/s13045-018-0689-y 30621731 PMC6325886

[60] Zhu J Jia X Xiao G Kang Y Partridge NC Qin L. EGF-like ligands stimulate osteoclastogenesis by regulating expression of osteoclast regulatory factors by osteoblasts: implications for osteolytic bone metastases. J Biol Chem (2007) 282(37):26656–64. 10.1074/jbc.M705064200 17636266

[61] Taverna S Pucci M Giallombardo M Di Bella MA Santarpia M Reclusa P. Amphiregulin contained in NSCLC-exosomes induces osteoclast differentiation through the activation of EGFR pathway. Sci Rep (2017) 7(1):3170. 10.1038/s41598-017-03460-y 28600504 PMC5466625

[62] Raimondo S Urzi O Conigliaro A Bosco GL Parisi S Carlisi M. Extracellular Vesicle microRNAs Contribute to the Osteogenic Inhibition of Mesenchymal Stem Cells in Multiple Myeloma. Cancers (2020) 12(2):449. 10.3390/cancers12020449 PMC7072478 32075123

[63] Zhang X Li R Qin X Wang L Xiao J Song Y. Sp1 Plays an Important Role in Vascular Calcification Both In Vivo and In Vitro. J Am Heart Assoc (2018) 7(6):e007555. 10.1161/JAHA.117.007555 29572322 PMC5907546

[64] Yu S Yerges-Armstrong LM Chu Y Zmuda JM Zhang Y. Transcriptional Regulation of Frizzled-1 in Human Osteoblasts by Sp1. PloS One (2016) 11(10):e0163277. 10.1371/journal.pone.0163277 27695039 PMC5047477

[65] Jafary F Hanachi P Gorjipour K. Osteoblast Differentiation on Collagen Scaffold with Immobilized Alkaline Phosphatase. Int J Organ Transplant Med (2017) 8(4):195–202. 29321835 PMC5756901

[66] Tiedemann K Sadvakassova G Mikolajewicz N Juhas M Sabirova Z Tabaries S. Exosomal Release of L-Plastin by Breast Cancer Cells Facilitates Metastatic Bone Osteolysis. Trans Oncol (2019) 12(3):462–74. 10.1016/j.tranon.2018.11.014 PMC6305809 30583289

[67] Tiedemann K Hussein O Sadvakassova G Guo Y Siegel PM Komarova SV. Breast cancer-derived factors stimulate osteoclastogenesis through the Ca2+/protein kinase C and transforming growth factor-beta/MAPK signaling pathways. J Biol Chem (2009) 284(48):33662–70. 10.1074/jbc.M109.010785 PMC2785208 19801662

[68] Rafiei S Tiedemann K Tabaries S Siegel PM Komarova SV. Peroxiredoxin 4: a novel secreted mediator of cancer induced osteoclastogenesis. Cancer Lett (2015) 361(2):262–70. 10.1016/j.canlet.2015.03.012 25779674

[69] Guo L Zhu Y Li L Zhou S Yin G Yu G. Breast cancer cell-derived exosomal miR-20a-5p promotes the proliferation and differentiation of osteoclasts by targeting SRCIN1. Cancer Med (2019) 8(12):5687–701. 10.1002/cam4.2454 PMC6745844 31385464

[70] Liu X Cao M Palomares M Wu X Li A Yan W. Metastatic breast cancer cells overexpress and secrete miR-218 to regulate type I collagen deposition by osteoblasts. Breast Cancer Res BCR (2018) 20(1):127. 10.1186/s13058-018-1059-y 30348200 PMC6198446

[71] Xu Z Liu X Wang H Li J Dai L Li J. Lung adenocarcinoma cell-derived exosomal miR-21 facilitates osteoclastogenesis. Gene (2018) 666:116–22. 10.1016/j.gene.2018.05.008 29730429

[72] Loh PG Yang HS Walsh MA Wang Q Wang X Cheng Z. Structural basis for translational inhibition by the tumour suppressor Pdcd4. EMBO J (2009) 28(3):274–85. 10.1038/emboj.2008.278 PMC2637334 19153607

[73] Tanaka S Nakamura K Takahasi N Suda T. Role of RANKL in physiological and pathological bone resorption and therapeutics targeting the RANKL-RANK signaling system. Immunol Rev (2005) 208:30–49. 10.1111/j.0105-2896.2005.00327.x 16313339

[74] Wang S Liu Z Wang J Ji X Yao Z Wang X. miR21 promotes osteoclastogenesis through activation of PI3K/Akt signaling by targeting Pten in RAW264.7 cells. Mol Med Rep (2020) 21(3):1125–32. 10.3892/mmr.2020.10938 PMC7003029 32016444

[75] Moon JB Kim JH Kim K Youn BU Ko A Lee SY. Akt induces osteoclast differentiation through regulating the GSK3beta/NFATc1 signaling cascade. J Immunol (2012) 188(1):163–9. 10.4049/jimmunol.1101254 22131333

[76] Ye Y Li SL Ma YY Diao YJ Yang L Su MQ. Exosomal miR-141-3p regulates osteoblast activity to promote the osteoblastic metastasis of prostate cancer. Oncotarget (2017) 8(55):94834–49. 10.18632/oncotarget.22014 PMC5706916 29212270

[77] Duan Y Tan Z Yang M Li J Liu C Wang C. PC-3-Derived Exosomes Inhibit Osteoclast Differentiation by Downregulating miR-214 and Blocking NF-kappaB Signaling Pathway. BioMed Res Int (2019) 2019:8650846. 10.1155/2019/8650846 31058194 PMC6463683

[78] McBeath R Pirone DM Nelson CM Bhadriraju K Chen CS. Cell shape, cytoskeletal tension, and RhoA regulate stem cell lineage commitment. Dev Cell (2004) 6(4):483–95. 10.1016/s1534-5807(04)00075-9 15068789

[79] Wang J Hendrix A Hernot S Lemaire M De Bruyne E Van Valckenborgh E. Bone marrow stromal cell-derived exosomes as communicators in drug resistance in multiple myeloma cells. Blood (2014) 124(4):555–66. 10.1182/blood-2014-03-562439 24928860

[80] Wang J De Veirman K De Beule N Maes K De Bruyne E Van Valckenborgh E. The bone marrow microenvironment enhances multiple myeloma progression by exosome-mediated activation of myeloid-derived suppressor cells. Oncotarget (2015) 6(41):43992–4004. 10.18632/oncotarget.6083 PMC4791281 26556857

[81] Roccaro AM Sacco A Maiso P Azab AK Tai YT Reagan M. BM mesenchymal stromal cell-derived exosomes facilitate multiple myeloma progression. J Clin Invest (2013) 123(4):1542–55. 10.1172/JCI66517 PMC3613927 23454749

[82] Colombo M Galletti S Bulfamante G Falleni M Tosi D Todoerti K. Multiple myeloma-derived Jagged ligands increases autocrine and paracrine interleukin-6 expression in bone marrow niche. Oncotarget (2016) 7(35):56013–29. 10.18632/oncotarget.10820 PMC5302893 27463014

[83] Coniglio SJ. Role of Tumor-Derived Chemokines in Osteolytic Bone Metastasis. Front Endocrinol (2018) 9:313. 10.3389/fendo.2018.00313 PMC5999726 29930538

[84] Ho M Chen T Liu J Dowling P Hideshima T Zhang L. Targeting histone deacetylase 3 (HDAC3) in the bone marrow microenvironment inhibits multiple myeloma proliferation by modulating exosomes and IL-6 trans-signaling. Leukemia (2020) 34(1):196–209. 10.1038/s41375-019-0493-x 31142847 PMC6883144

[85] Roccaro AM Sacco A Thompson B Leleu X Azab AK Azab F. MicroRNAs 15a and 16 regulate tumor proliferation in multiple myeloma. Blood (2009) 113(26):6669–80. 10.1182/blood-2009-01-198408 PMC2710922 19401561

[86] Swarbrick A Woods SL Shaw A Balakrishnan A Phua Y Nguyen A. miR-380-5p represses p53 to control cellular survival and is associated with poor outcome in MYCN-amplified neuroblastoma. Nat Med (2010) 16(10):1134–40. 10.1038/nm.2227 PMC3019350 20871609

[87] Bilen MA Pan T Lee YC Lin SC Yu G Pan J. Proteomics Profiling of Exosomes from Primary Mouse Osteoblasts under Proliferation versus Mineralization Conditions and Characterization of Their Uptake into Prostate Cancer Cells. J Proteome Res (2017) 16(8):2709–28. 10.1021/acs.jproteome.6b00981 PMC5860883 28675788

[88] Morhayim J van de Peppel J Dudakovic A Chiba H van Wijnen AJ van Leeuwen JP. Molecular characterization of human osteoblast-derived extracellular vesicle mRNA using next-generation sequencing. Biochim Biophys Acta Mol Cell Res (2017) 1864(7):1133–41. 10.1016/j.bbamcr.2017.03.011 PMC5639703 28347747

[89] Morhayim J van de Peppel J Demmers JA Kocer G Nigg AL van Driel M. Proteomic signatures of extracellular vesicles secreted by nonmineralizing and mineralizing human osteoblasts and stimulation of tumor cell growth. FASEB J Off Publ Fed Am Societies Exp Biol (2015) 29(1):274–85. 10.1096/fj.14-261404 25359493

[90] Cui Y Luan J Li H Zhou X Han J. Exosomes derived from mineralizing osteoblasts promote ST2 cell osteogenic differentiation by alteration of microRNA expression. FEBS Lett (2016) 590(1):185–92. 10.1002/1873-3468.12024 26763102

[91] Lener T Gimona M Aigner L Borger V Buzas E Camussi G. Applying extracellular vesicles based therapeutics in clinical trials - an ISEV position paper. J Extracellular Vesicles (2015) 4:30087. 10.3402/jev.v4.30087 26725829 PMC4698466

[92] Im EJ Lee CH Moon PG Rangaswamy GG Lee B Lee JM. Sulfisoxazole inhibits the secretion of small extracellular vesicles by targeting the endothelin receptor A. Nat Commun (2019) 10(1):1387. 10.1038/s41467-019-09387-4 30918259 PMC6437193

[93] Bobrie A Krumeich S Reyal F Recchi C Moita LF Seabra MC. Rab27a supports exosome-dependent and -independent mechanisms that modify the tumor microenvironment and can promote tumor progression. Cancer Res (2012) 72(19):4920–30. 10.1158/0008-5472.CAN-12-0925 22865453

[94] Kosaka N Iguchi H Yoshioka Y Takeshita F Matsuki Y Ochiya T. Secretory mechanisms and intercellular transfer of microRNAs in living cells. J Biol Chem (2010) 285(23):17442–52. 10.1074/jbc.M110.107821 PMC2878508 20353945

[95] Fabbri M Paone A Calore F Galli R Gaudio E Santhanam R. MicroRNAs bind to Toll-like receptors to induce prometastatic inflammatory response. Proc Natl Acad Sci U States America (2012) 109(31):E2110–6. 10.1073/pnas.1209414109 PMC3412003 22753494

[96] Faict S Muller J De Veirman K De Bruyne E Maes K Vrancken L. Exosomes play a role in multiple myeloma bone disease and tumor development by targeting osteoclasts and osteoblasts. Blood Cancer J (2018) 8(11):105. 10.1038/s41408-018-0139-7 30409995 PMC6224554

[97] Khan FM Saleh E Alawadhi H Harati R Zimmermann WH El-Awady R. Inhibition of exosome release by ketotifen enhances sensitivity of cancer cells to doxorubicin. Cancer Biol Ther (2018) 19(1):25–33. 10.1080/15384047.2017.1394544 29244610 PMC5790333

[98] Nishida-Aoki N Tominaga N Takeshita F Sonoda H Yoshioka Y Ochiya T. Disruption of Circulating Extracellular Vesicles as a Novel Therapeutic Strategy against Cancer Metastasis. Mol Ther J Am Soc Gene Ther (2017) 25(1):181–91. 10.1016/j.ymthe.2016.10.009 PMC5363297 28129113

[99] Baglio SR Lagerweij T Perez-Lanzon M Ho XD Leveille N Melo SA. Blocking Tumor-Educated MSC Paracrine Activity Halts Osteosarcoma Progression. Clin Cancer Res an Off J Am Assoc Cancer Res (2017) 23(14):3721–33. 10.1158/1078-0432.CCR-16-2726 28053020

[100] Hoshino A Costa-Silva B Shen TL Rodrigues G Hashimoto A Tesic Mark M. Tumour exosome integrins determine organotropic metastasis. Nature (2015) 527(7578):329–35. 10.1038/nature15756 PMC4788391 26524530

[101] Morikawa S Mabuchi Y Kubota Y Nagai Y Niibe K Hiratsu E. Prospective identification, isolation, and systemic transplantation of multipotent mesenchymal stem cells in murine bone marrow. J Exp Med (2009) 206(11):2483–96. 10.1084/jem.20091046 PMC2768869 19841085

[102] Qin Y Wang L Gao Z Chen G Zhang C. Bone marrow stromal/stem cell-derived extracellular vesicles regulate osteoblast activity and differentiation in vitro and promote bone regeneration in vivo. Sci Rep (2016) 6:21961. 10.1038/srep21961 26911789 PMC4766421

[103] Zhang J Liu X Li H Chen C Hu B Niu X. Exosomes/tricalcium phosphate combination scaffolds can enhance bone regeneration by activating the PI3K/Akt signaling pathway. Stem Cell Res Ther (2016) 7(1):136. 10.1186/s13287-016-0391-3 27650895 PMC5028974

[104] Tian Y Li S Song J Ji T Zhu M Anderson GJ. A doxorubicin delivery platform using engineered natural membrane vesicle exosomes for targeted tumor therapy. Biomaterials (2014) 35(7):2383–90. 10.1016/j.biomaterials.2013.11.083 24345736

[105] Pascucci L Cocce V Bonomi A Ami D Ceccarelli P Ciusani E. Paclitaxel is incorporated by mesenchymal stromal cells and released in exosomes that inhibit in vitro tumor growth: a new approach for drug delivery. J Controlled Release Off J Controlled Release Soc (2014) 192:262–70. 10.1016/j.jconrel.2014.07.042 25084218

[106] Kim MS Haney MJ Zhao Y Yuan D Deygen I Klyachko NL. Engineering macrophage-derived exosomes for targeted paclitaxel delivery to pulmonary metastases: in vitro and in vivo evaluations. Nanomed Nanotechnol Biol Med (2018) 14(1):195–204. 10.1016/j.nano.2017.09.011 28982587

[107] Kim MS Haney MJ Zhao Y Mahajan V Deygen I Klyachko NL. Development of exosome-encapsulated paclitaxel to overcome MDR in cancer cells. Nanomed Nanotechnol Biol Med (2016) 12(3):655–64. 10.1016/j.nano.2015.10.012 PMC4809755 26586551

[108] Garofalo M Villa A Rizzi N Kuryk L Rinner B Cerullo V. Extracellular vesicles enhance the targeted delivery of immunogenic oncolytic adenovirus and paclitaxel in immunocompetent mice. J Controlled Release Off J Controlled Release Soc (2019) 294:165–75. 10.1016/j.jconrel.2018.12.022 30557650

[109] Melzer C Ohe JV Hass R. Anti-Tumor Effects of Exosomes Derived from Drug-Incubated Permanently Growing Human MSC. Int J Mol Sci (2020) 21(19):7311. 10.3390/ijms21197311 PMC7582671 33023058

[110] Wei H Chen J Wang S Fu F Zhu X Wu C. A Nanodrug Consisting Of Doxorubicin And Exosome Derived From Mesenchymal Stem Cells For Osteosarcoma Treatment In Vitro. Int J Nanomed (2019) 14:8603–10. 10.2147/IJN.S218988 PMC6830377 31802872

[111] Cappariello A Loftus A Muraca M Maurizi A Rucci N Teti A. Osteoblast-Derived Extracellular Vesicles Are Biological Tools for the Delivery of Active Molecules to Bone. J Bone Mineral Res Off J Am Soc Bone Mineral Res (2018) 33(3):517–33. 10.1002/jbmr.3332 29091316

[112] Liu KD Cao NJ Zhu YH Wang W. Exosome: A Novel Nanocarrier Delivering Noncoding RNA for Bone Tissue Engineering. J Nanomater (2020) 2020:2187169. 10.1155/2020/2187169

[113] Alvarez-Erviti L Seow Y Yin H Betts C Lakhal S Wood MJ. Delivery of siRNA to the mouse brain by systemic injection of targeted exosomes. Nat Biotechnol (2011) 29(4):341–5. 10.1038/nbt.1807 21423189

[114] Wang C Chen L Huang Y Li K Jinye A Fan T. Exosome-delivered TRPP2 siRNA inhibits the epithelial-mesenchymal transition of FaDu cells. Oncol Lett (2019) 17(2):1953–61. 10.3892/ol.2018.9752 PMC6341906 30675260

[115] Hesse E Taipaleenmaki H. MicroRNAs in Bone Metastasis. Curr Osteoporosis Rep (2019) 17(3):122–8. 10.1007/s11914-019-00510-4 30905007

[116] Katakowski M Buller B Zheng X Lu Y Rogers T Osobamiro O. Exosomes from marrow stromal cells expressing miR-146b inhibit glioma growth. Cancer Lett (2013) 335(1):201–4. 10.1016/j.canlet.2013.02.019 PMC3665755 23419525

[117] Valencia K Luis-Ravelo D Bovy N Anton I Martinez-Canarias S Zandueta C. miRNA cargo within exosome-like vesicle transfer influences metastatic bone colonization. Mol Oncol (2014) 8(3):689–703. 10.1016/j.molonc.2014.01.012 24593875 PMC5528646

[118] Wei J Li H Wang S Li T Fan J Liang X. let-7 enhances osteogenesis and bone formation while repressing adipogenesis of human stromal/mesenchymal stem cells by regulating HMGA2. Stem Cells Dev (2014) 23(13):1452–63. 10.1089/scd.2013.0600 PMC4066225 24617339

[119] Khani AT Sharifzad F Mardpour S Hassan ZM Ebrahimi M. Tumor extracellular vesicles loaded with exogenous Let-7i and miR-142 can modulate both immune response and tumor microenvironment to initiate a powerful anti-tumor response. Cancer Lett (2020) 501:200–9. 10.1016/j.canlet.2020.11.014 33220334

[120] Ohno S Takanashi M Sudo K Ueda S Ishikawa A Matsuyama N. Systemically injected exosomes targeted to EGFR deliver antitumor microRNA to breast cancer cells. Mol Ther J Am Soc Gene Ther (2013) 21(1):185–91. 10.1038/mt.2012.180 PMC3538304 23032975

[121] Hong CS Sharma P Yerneni SS Simms P Jackson EK Whiteside TL. Circulating exosomes carrying an immunosuppressive cargo interfere with cellular immunotherapy in acute myeloid leukemia. Sci Rep (2017) 7(1):14684. 10.1038/s41598-017-14661-w 29089618 PMC5666018

[122] Danilin S Merkel AR Johnson JR Johnson RW Edwards JR Sterling JA. Myeloid-derived suppressor cells expand during breast cancer progression and promote tumor-induced bone destruction. Oncoimmunology (2012) 1(9):1484–94. 10.4161/onci.21990 PMC3525604 23264895

